# Spatial decision on allocating automated external defibrillators (AED) in communities by multi-criterion two-step floating catchment area (MC2SFCA)

**DOI:** 10.1186/s12942-016-0046-8

**Published:** 2016-05-25

**Authors:** Bo-Cheng Lin, Chao-Wen Chen, Chien-Chou Chen, Chiao-Ling Kuo, I-chun Fan, Chi-Kung Ho, I-Chuan Liu, Ta-Chien Chan

**Affiliations:** Research Center for Humanity and Social Sciences, Academia Sinica, 128 Academia Road, Section 2, Nankang, Taipei, 115 Taiwan; Division of Trauma, Department of Surgery, Kaohsiung Medical University Hospital, Kaohsiung, Taiwan; Department of Emergency Medicine, Kaohsiung Medical University Hospital, Kaohsiung Medical University, 100 Tzyou 1st Road, Kaohsiung, 807 Taiwan; Department of Geomatics, National Cheng Kung University, Tainan, Taiwan; Institute of History and Philology, Academia Sinica, Taipei, Taiwan; Department of Health, Kaohsiung City Government, Kaohsiung, Taiwan; Department of Public Health, Kaohsiung Medical University, Kaohsiung, Taiwan; Fire Bureau, Kaohsiung City Government, Kaohsiung, Taiwan

**Keywords:** Out-of-hospital cardiac arrest, Zero-inflated Poisson model, Basic statistical area, Bayesian analysis, Priority ranking

## Abstract

**Background:**

The occurrence of out-of-hospital cardiac arrest (OHCA) is a critical life-threatening event which frequently warrants early defibrillation with an automated external defibrillator (AED). The optimization of allocating a limited number of AEDs in various types of communities is challenging. We aimed to propose a two-stage modeling framework including spatial accessibility evaluation and priority ranking to identify the highest gaps between demand and supply for allocating AEDs.

**Methods:**

In this study, a total of 6135 OHCA patients were defined as demand, and the existing 476 publicly available AEDs locations and 51 emergency medical service (EMS) stations were defined as supply. To identify the demand for AEDs, Bayesian spatial analysis with the integrated nested Laplace approximation (INLA) method is applied to estimate the composite spatial risks from multiple factors. The population density, proportion of elderly people, and land use classifications are identified as risk factors. Then, the multi-criterion two-step floating catchment area (MC2SFCA) method is used to measure spatial accessibility of AEDs between the spatial risks and the supply of AEDs. Priority ranking is utilized for prioritizing deployment of AEDs among communities because of limited resources.

**Results:**

Among 6135 OHCA patients, 56.85 % were older than 65 years old, and 79.04 % were in a residential area. The spatial distribution of OHCA incidents was found to be concentrated in the metropolitan area of Kaohsiung City, Taiwan. According to the posterior mean estimated by INLA, the spatial effects including population density and proportion of elderly people, and land use classifications are positively associated with the OHCA incidence. Utilizing the MC2SFCA for spatial accessibility, we found that supply of AEDs is less than demand in most areas, especially in rural areas. Under limited resources, we identify priority places for deploying AEDs based on transportation time to the nearest hospital and population size of the communities.

**Conclusion:**

The proposed method will be beneficial for optimizing resource allocation while considering multiple local risks. The optimized deployment of AEDs can broaden EMS coverage and minimize the problems of the disparity in urban areas and the deficiency in rural areas.

## Background

The occurrence of out-of-hospital cardiac arrest (OHCA) is a critical life-threatening event throughout the world [1, 2]. Approximately 424,000 persons experience an OHCA each year in the United States [3], as do 275,000 in Europe [4], 60,000 in Japan [5] and 20,000 in Taiwan. Half of OHCA patients have no symptoms before the onset of arrest, and the survival rate is below 10 % [6]. Thus, the concept of the chain of survival is introduced to improve the chance of surviving OHCA [7]. In the chain of survival, prompt cardiopulmonary resuscitation (CPR) and early defibrillation are the primary factors in determining the survival from OHCA [8, 9]. Without CPR and defibrillation, the survival rate decreases by 7–10 %/min of delay [10].

As most cardiac arrest patients are experiencing ventricular fibrillation (VF) [11], early defibrillation with the use of an automated external defibrillator (AED) is the primary determinant of survival. The AED is a device that can be easily used by nonprofessional rescuers. Therefore, the American Heart Association (AHA) has promoted the concept of publicly accessible AEDs [12, 13], and the deployment of AEDs has also become an important part of emergency medical systems (EMS).

In Taiwan, the Department of Health (DOH) has implemented a plan to make AEDs available in public places since 2013 under the Emergency Medical Services Act [14]. Currently, AEDs have been placed in public locations such as railway stations, shopping centers, schools and hotels. However, previous studies have shown that most cases of OHCA occur in residential areas [15, 16]. This causes a gap between the placement of AEDs and the locations with most frequent OHCA events, but the issue of how to allocate AEDs efficiently in communities is seldom discussed. To deploy a limited number of AEDs with high spatial accessibility, it is warranted to consider the spatial optimization of AEDs, especially in residential communities. Extending the coverage of AEDs will help enhance the timeliness of first-aid resuscitation and also expand the service areas of traditional EMS.

Spatial accessibility refers to the probable utilization of a service location, and is determined by the spatial distribution between supply and demand. The two-step floating catchment area (2SFCA) is a popular method and has been widely used in measurement of healthcare accessibility [17–21]. The 2SFCA is a special case of the gravity-based measure [22] and is implemented in a two-step process to compute supply-to-demand ratio at each demand location. However, the 2SFCA assumes that all locations have equal access to supply locations within a catchment and no access to supply locations outside of the catchment. To address these shortcomings, an enhanced 2SFCA (E2SFCA) method [18] is proposed to introduce weights to differentiate travel time zones to account for stepwise decaying of accessibility within each catchment. Furthermore, the distance decay is improved to a continuous function by involving a kernel density [23] or a Gaussian function [24]. The service area of a catchment in 2SFCA also needs to vary based on the type of supply [25]. A variable 2SFCA (V2SFCA) method [26] is proposed to introduce an adaptive size of the catchment determined according to the service capacity of the supply location. To consider the overestimation of demand on supply locations in the E2SFCA, the competition among supply locations is introduced in a three-step floating catchment area (3SFCA) method [27]. The 3SFCA assumes that probable demand is influenced by the availability of other nearby supply locations.

Before the location allocation analysis, the demand needs to be identified first. Demographic characteristics such as population density, age, gender, income and education were related to higher frequency of OHCA events in previous studies [16, 28, 29]. Demand should therefore best assessed by a composite indicator rather than a single factor. Thus, we applied a regression model to account for multiple influencing factors, and estimate the potential risk as a demand factor in the computation of spatial accessibility. The supply factor is measured based on registered AEDs and EMS. The placement of AEDs can then be identified according to a two-stage modeling framework including spatial accessibility evaluation and priority ranking in this study. As for location allocation analysis, spatial accessibility needs to take into consideration both supply and demand [30, 31]. We aim to identify the gaps between demand and supply of AEDs and prioritize the order of urgency geographically.

## Methods

### Ethics

The study was approved by the committee of the Institutional Review Board (IRB) at Academia Sinica (AS-IRB01-14013). The databases we used were all stripped of identifying information and thus informed consent was not needed.

### Data

The data used in this study can be divided into two categories including OHCA, and spatial and statistical data. This was a 3-year retrospective study during 2011–2013 from a cardiac arrest cohort in the city of Kaohsiung, Taiwan. Kaohsiung covers 2947 km^2^ including both urban and rural communities. The total population of Kaohsiung was 2.64 million in 2012. The records of OHCA patients were obtained from the DOH, Kaohsiung City Government. We enrolled all patients who suffered an OHCA and were treated by emergency medical technicians (EMTs) from January 1, 2011 to December 31, 2013. Cardiac arrest was defined as the abrupt loss of heart function in a person as confirmed by EMTs. Their corresponding onset locations were geocoded by using the position service provided by the Ministry of the Interior, Taiwan.

Spatial data include the polygon of basic statistical areas (BSAs), land use data, and the location of existing AEDs and EMS stations. The BSA was developed by the Statistics Department of the Ministry of the Interior in 2012. It is the basic spatial unit for official statistics. The major concerns are protection of privacy and size of the unit in terms of geographical space, the stability of the statistical units and attribute consideration [32]. There are 17,389 BSAs in Kaohsiung City. The land use data are derived from the National Land Surveying and Mapping Center, Ministry of the Interior (http://lui.nlsc.gov.tw/LUWeb/). The latest update on the investigation of the land use data is December 2014, and the spatial scale is 1:5000. The land use data are classified into a three-level hierarchical structure. There are nine categories including agriculture, forest, transportation, water conservation, built-up land, public, amusement and rest, rock salt and others in the first level. The location of 476 AEDs was derived from the Taiwan Public AED Registry website of the Ministry of Health and Welfare in October 2014 (http://tw-aed.mohw.gov.tw/). In addition, we also include 51 EMS stations in Kaohsiung City. The statistical data contain the total and 65-and-older population of each BSA as of June 2012, and were downloaded from the Department of Statistics of Taiwan (http://segis.moi.gov.tw/STAT/).

### Data processing

**Fig. 1:**
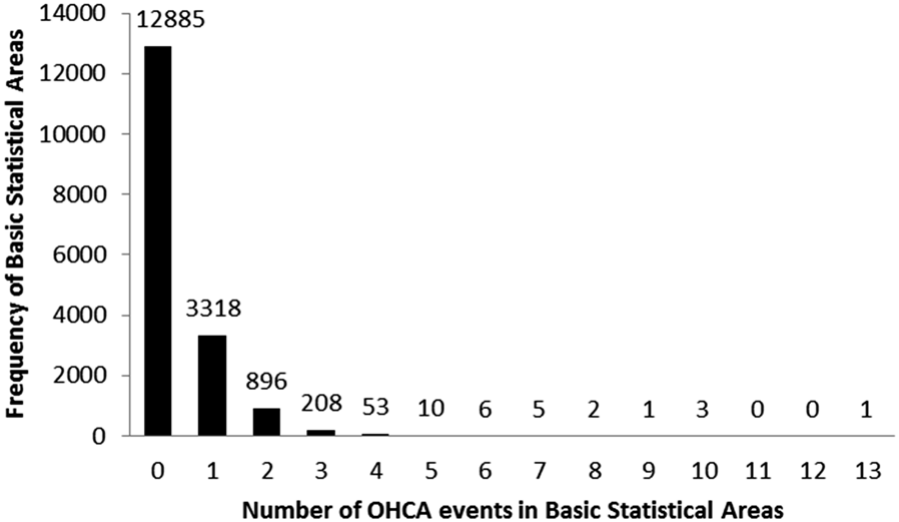
Distribution of OHCA events in Kaohsiung City from 2011 to 2013

Among 17,389 BSAs, there is one extreme value of population density which is 195 times higher than the average. To avoid inference bias from this outlier, we decided to remove this BSA from our whole analysis. Therefore, there are 17,388 BSAs included in this study. The onset locations of OHCA events were geocoded into BSAs. Due to missing or vague addresses, we excluded 308 OHCA events (4.6 %) which were unable to be geocoded. In addition, the OHCA events located in nursing homes were also excluded. Since nursing homes are a kind of long-term care facility, appropriate medical equipment and AED are always installed. We treated these cases as in-hospital cardiac arrest, not cases occurring in communities. The final number of observations for allocation analysis was 6135. After geocoding and using the spatial join function of ArcGIS 10.2 (ESRI Inc., Redlands, CA, USA), the OHCA events in each BSA can be counted. For an overview of the frequencies of OHCA events, a histogram is given in Fig. 1. It shows that the OHCA events exhibit a large number of zeros in some BSAs. Thus, we need to choose a zero-inflated Poisson (ZIP) model to deal with this situation. From the first level of land use data, the categories of transportation (including airports, railroads, roads and harbors) and public (including governmental agencies, schools, medical health care facilities, social welfare facilities and public utilities) are considered risk factors, since the transportation and public are categories of population clusters in the daytime. OHCA events also often occur in the transportation and public categories. In the second level from the built-up land, there are four subcategories, including commerce, housing, industry and others. Only the housing subcategory is used, since the community is the main consideration in this analysis. The housing category contains pure housing as well as residential buildings which are also partly used for industry and trade. Thus the content in the housing subcategory contains mixed uses. And the percentages of housing, transportation and public area in each BSA are calculated by ArcGIS using the intersection and frequency functions on the land use and BSA map. The demographic characteristics including the population density and the percentage of 65-and-over population were also calculated based on each BSA.

### The multi-criterion two-step floating catchment area (MC2SFCA)

The multi-criterion two-step floating catchment area (MC2SFCA) is extended from the 2SFCA [22], which also considers interaction between demand and supply within a region. In MC2SFCA, the demand is accessed by a composite indicator rather than a single factor. The composite indicator is regarded as the potential risk which can be estimated by the regression model with multiple influencing factors. The regression modeling is implemented by spatial regression analysis to understand the relationship between OHCA incidence and possible risk factors. There are two kinds of risk factors considered in the regression model. One is demographic characteristics including population density and the proportion of 65-and-older population, and the other is the percentage of land use classification including transportation, and public and housing areas. To analyze OHCA count data with excess zeros, the most well-known model is ZIP regression [33]. The ZIP model is a mixture of Poisson and degenerate distributions. This model assumes that the zero count consists of two types. The first one occurs with probability *p* and the observation is only zero, while the other occurs with probability (1 − *p*) according to a Poisson distribution. We used Bayesian analysis of the spatial count data with the ZIP model to estimate and consider the spatial variability of their relationships based on BSA level.

In Bayesian statistics, unknown quantities are treated as random variables and can be described in terms of probability distributions [34]. This provides a more intuitive quantity rather than a fixed value in frequentist statistics. Our spatial regression model can be represented as follow:

$$ y_{i} = \alpha + \mathop \sum \limits_{m = 1}^{5} \beta_{m} x_{mi} + u_{i} + v_{i} $$

*y*_*i*_ was the number of OHCA incidents observed in the ith BSA, *α* is the model intercept, and the regression coefficients $\varvec{\beta}= (\beta_{1} , \ldots ,\beta_{5} )$ quantify the effect of risk factors stored in $\varvec{x} = (x_{1} , \ldots ,x_{5} ).$*u*_*i*_ and *v*_*i*_ denote spatial and independent error terms respectively. Thus, the purpose of the Bayesian statistics is to derive the posterior distribution of parameters of interest. The Bayesian approach is increasingly adopted in spatial analysis [35–37], since it allows use of complicated models for spatial correlation and use of Markov chain Monte Carlo (MCMC) simulation algorithms [34]. However, there remain computational challenges in Bayesian computation for complicated probability models, such as spatial data [38]. To improve the computational efficiency of Bayesian analysis, the integrated nested Laplace approximation (INLA) approach has been developed to compute a fast and accurate approximation to the posterior distributions of the parameters in latent Gaussian models [39]. It can be implemented by an R package named R-INLA (http://www.r-inla.org/). After regression of spatial count data with R-INLA, the posterior distribution of OHCA incidence and its coefficients can be derived. The mean value of the posterior distribution of OHCA incidence is used as the risk value for the MC2SFCA method.

After regression modeling, the potential risk is used as the demand factor in the computation of spatial accessibility. Spatial accessibility estimates the activities between locations in space, and thus is a classic issue for location analysis [31]. To evaluate AED accessibility, a 2SFCA [22] is used. The floating catchment area method defines the service area of a location based on a threshold distance of travel time. The 2SFCA repeats the process of floating catchment twice, once on supply locations and once on demand locations. For demand locations, the risk values are derived from the result of R-INLA. For supply sites, two kinds of data with different service areas are considered. They are the existing AEDs and ambulances of EMS stations. To reach an existing AED, a witness has to go and return to the location of an OHCA patient, but an ambulance is dispatched from an EMS station directly to the location of an OHCA patient. Thus, the service areas for the existing AEDs and ambulances are different during the same period. Since the use of AED within 4 min of cardiac arrest has been demonstrated to improve survival [40], the serving distance is 200 m for the existing AED and 3600 m for EMS based on the speed for an adult walking and ambulance speeds between about 100 m/min [41] and 900 m/min [42] respectively. With increasing time to treat with AED, the chance of survival among OHCA patients is decreased. Thus, we also incorporated a continuously function as distance decay in the 2SFCA to represent relative spatial access to AED. The Gaussian function proposed by Dai [24] is adopted as the decay function. The 2SFCA with Gaussian function is implemented in two steps. The first step is to search all demand locations *k* within the catchment area of supply location *j*, and the supply-to-demand ratio *R*_*j*_ of each supply location can be calculated by

$$ R_{j} = \frac{{S_{j} }}{{\mathop \sum \nolimits_{{k \in \{ d_{kj} \le d_{0} \} }} D_{k} G\left( {d_{kj} ,d_{0} } \right)}} $$

where *S*_*j*_ is the capacity of supply at *j*, and *D*_*k*_ is the demand at location *k* within the catchment area, *d*_0_ is the catchment size, and *d*_*kj*_ is the distance between demand location *k* and supply location *j*. *G* is the distance decay function based on the Gaussian function which is a smooth decay with increasing distance and can be formulated as [24]:

$$ G\left({d_{kj},d_{0}} \right) = \left\{{\begin{array}{*{20}l} {\frac{{e^{{- 1/2 \times \left({d_{kj}/d_{0}} \right)^{2}}} - e^{- 1/2}}}{{1 - e^{- 1/2}}},} \hfill &\quad {d_{kj} \le d_{0}} \hfill \\ {0,} \hfill &\quad {d_{kj} > d_{0}} \hfill \\ \end{array}} \right. $$

The second step is to search all supply locations *j* that fall inside the catchment area of demand location *i*, and determine the spatial accessibility by summing up the supply-to-demand ratio *R*_*j*_ weighted with the distance decay function *G*. Then, the spatial accessibility at demand location *i*, *A*_*i*_, is given by

$$ A_{i} = \mathop \sum \limits_{{j \in \{ d_{ij} \le d_{0} \} }} R_{j} G\left( {d_{ij} ,d_{0} } \right) $$

In the 2SFCA process, a supply-to-demand ratio is first estimated for each AED and EMS location. The total demand of each AED and EMS location is determined by the sum of risk value in the BSAs whose centroid falls within the 4-min arrival time catchment. In the second step, spatial accessibility of AED in each BSA is obtained by summing up the supply-to-demand ratios of AED and EMS locations within the catchment of the BSA.

### Priority ranking

The spatial accessibility can be evaluated by processing the MC2SFCA method in the previous section. Then, the spatial allocation of AEDs can be determined based on the values of spatial accessibility of AEDs in each BSA. However, AEDs could not be deployed in all of the BSA with lower accessibility in practice because of limited resources. Ranking of priority among the BSAs with lower access to AED is needed. It is assumed that an OHCA patient can be sent to the nearest hospital within 4 min. Among the BSAs with lower accessibility, the BSAs for which transportation time from the centroid of BSA to a nearest hospital is within 4 min are assigned a lower ranking. If the transportation time from the BSA centroid to the nearest hospital is over 4 min, the second priority ranking is based on the number of population in the BSA. In summary, the BSAs with longer transportation time to the hospital and with higher population are the first priority to deploy AEDs.

## Results

**Fig. 2:**
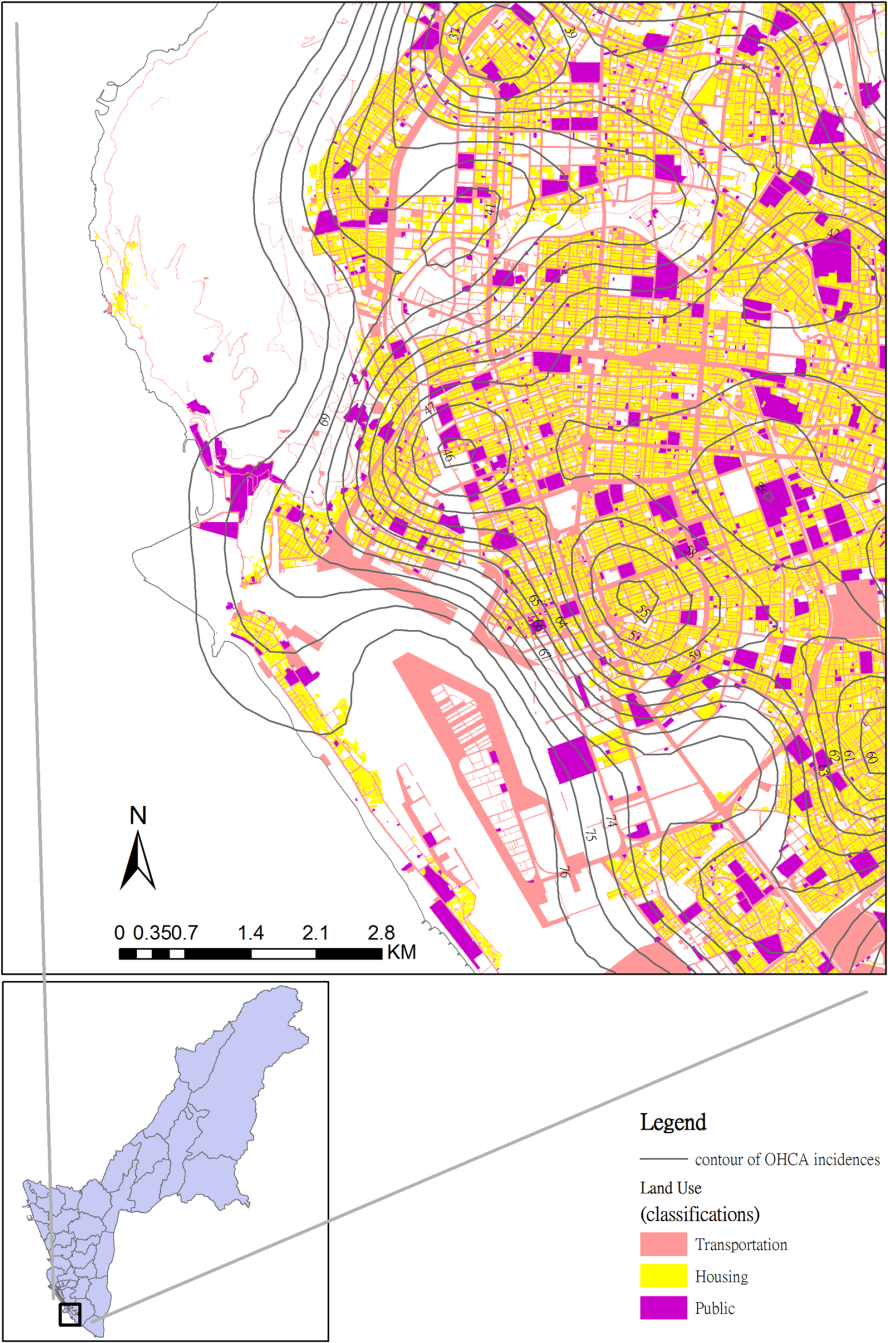
*Contour* map of OHCA incidents on different categories of land use in Kaohsiung City

As Table 1 shows, among 6135 OHCA patients, 3488 (56.85 %) were older than 65 years old, and 4849 (79.04 %, Table 2) were in a residential area. Aside from residential areas, there were also 559 (9.11 %) and 167 (2.72 %) patients using transportation and in public areas respectively (Table 2). In Fig. 2, contour lines identify areas with a greater number of OHCA events, and the map shows three different land use classifications including built-up land, transportation and public areas. It shows that the OHCA events were found to be clustering in southern Kaohsiung, which is the urban area.

**Table 1:** Age distribution of OHCA patients

Age (years)	N
0-14	65 (1.07 %)
15–64	2494 (40.65 %)
65+	3488 (56.85 %)
Unknown	88 (1.43 %)
Total	6135

**Table 2:** Distribution of OHCA incidents by land use classification

Classification	N
Agriculture	34 (0.55 %)
Forest	8 (0.13 %)
Transportation	559 (9.11 %)
Water conservation	14 (0.23 %)
Built-up land	5240 (85.41 %)
Trade	213
Housing	4849
Industry	121
Other structural purpose places	57
Public	167 (2.72 %)
Amusement and rest	75 (1.22) %
Rock salt	0 (0.00 %)
Other	38 (0.62 %)
Total	6135

**Fig. 3:**
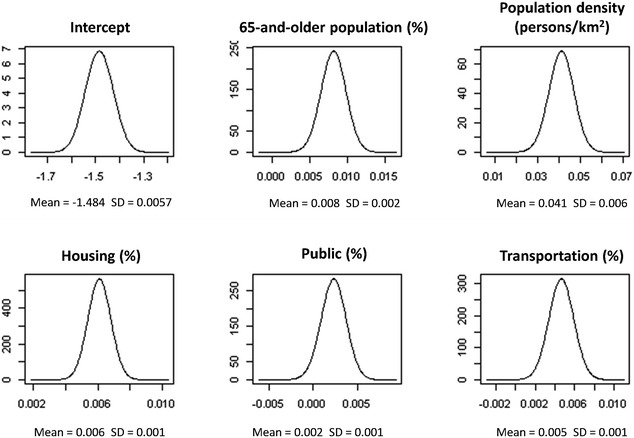
Posterior density for fixed effects of the risk model; the spatial risk factors are the proportion of 65-and-older population, population density, and the percentages of housing, transformation and public categories in BSA

**Fig. 4:**
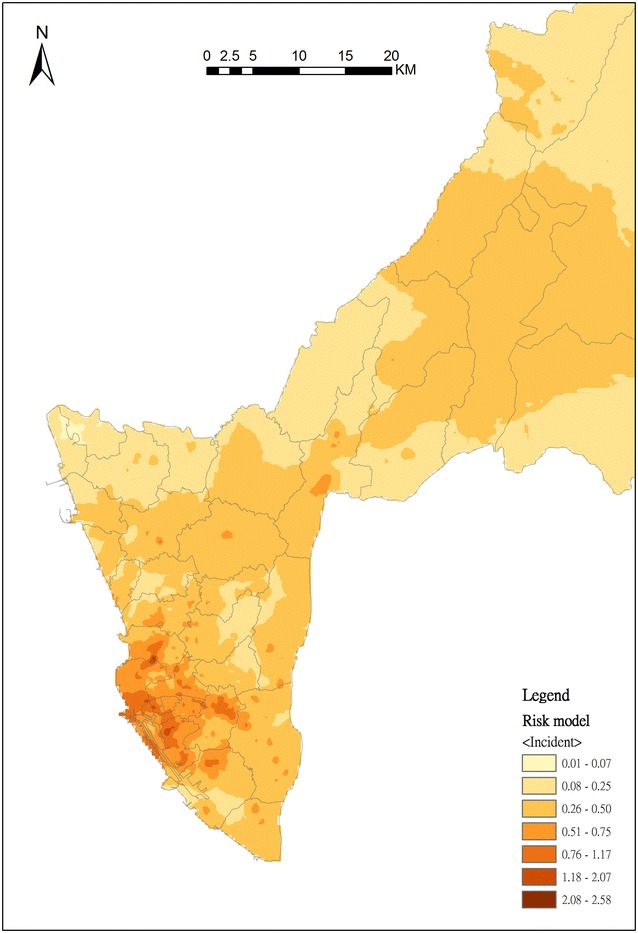
Geographical distribution of risk adjusted by spatial risk factors including the proportion of 65-and-older population, population density, and the percentages of housing, transformation and public categories in BSA

**Fig. 5:**
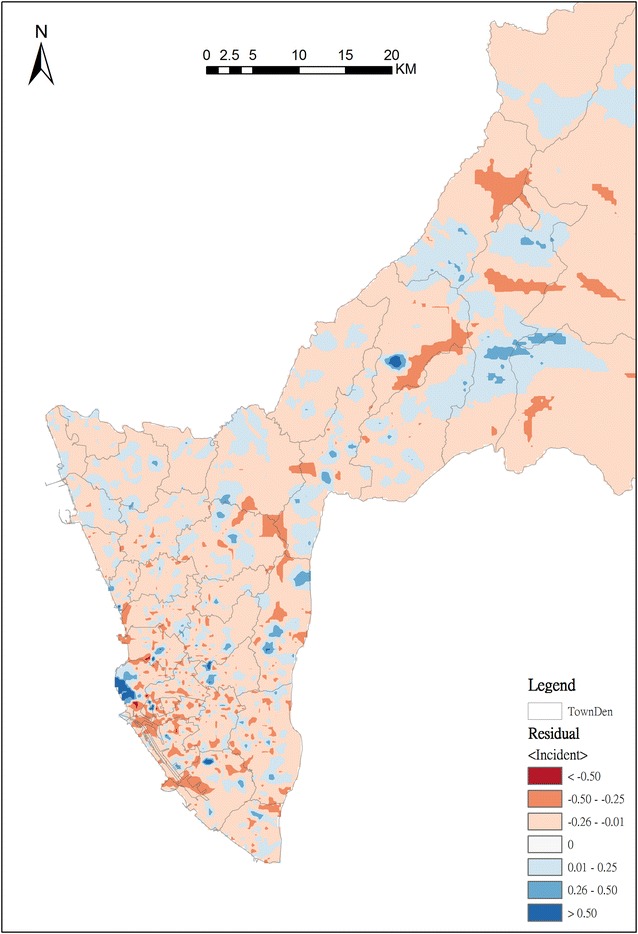
The residual map between the OHCA incidents and the estimated mean of posterior distribution derived from R-INLA

Next, we applied the R-INLA to identify the effect of risk factors and possible clusters. After adjusting by two demographic characteristics—population density and proportion of elderly people—and three land use classifications—transportation, housing and public—the posterior density plots for spatial effects of the risk model are shown in Fig. 3. This indicates that the spatial effects including population density, the proportion of 65-and-older population, and the percentage of transportation, public and housing area are positively associated with the OHCA incidence according to the result of posterior means. The posterior means of OHCA incidents in each BSA for 2011–2013 are mapped (Fig. 4). The OHCA incidents were found to be concentrated in southwestern Kaohsiung City, which was the metropolitan area, in contrast with the northeastern rural and mountainous areas of Kaohsiung City, where OHCA incidents are rare. To evaluate the fit of the Bayesian spatial regression, the residuals between the observed OHCA events and the expected values are shown on the residual map (Fig. 5). The global Moran’s I index was 0.0005 (p = 0.32) for the residual map. The spatial autocorrelation of the residuals was not statistically significant, which indicates there was no global spatial clustering after Bayesian spatial regression. The areas marked with dark blue and red represent the highest difference. Areas with dark blue indicate that the observed OHCA events are greater than the expected values, while areas with dark red show that the observed OHCA events are fewer than expected.

**Fig. 6:**
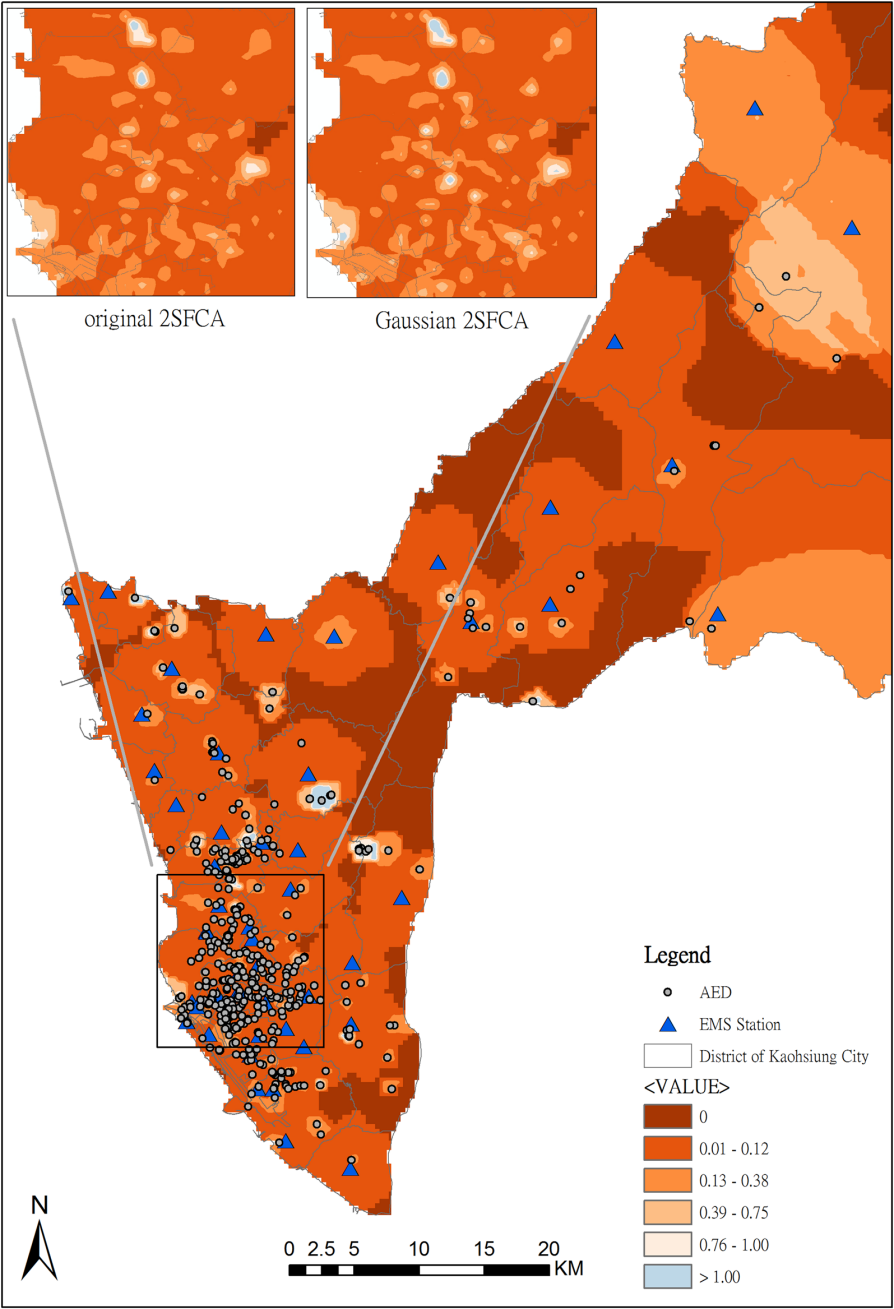
Accessibility of onsite and dispatched AED services in Kaohsiung City by MC2SFCA

We then used the risk model as our demand, and the deployed AED as our supply. The spatial accessibility is evaluated by 2SFCA with the Gaussian function, and the result is shown in Fig. 6. Locations where the AED supply is less than demand is marked with red color when the accessibility value is lower than 1. The accessibility of deployed AED in most areas is lower than 1, especially the areas in northern Kaohsiung City, which are the rural and mountainous area. From a comparison to the original 2SFCA, we found that the pattern is similar throughout the whole of Kaohsiung city. At a large scale in the urban area, the accessibility measure by Gaussian 2SFCA is smoother than the original 2SFCA. Counting the zero accessibility, the number increases from 2082 to 2109 after the Gaussian 2SFCA is used.

**Fig. 7:**
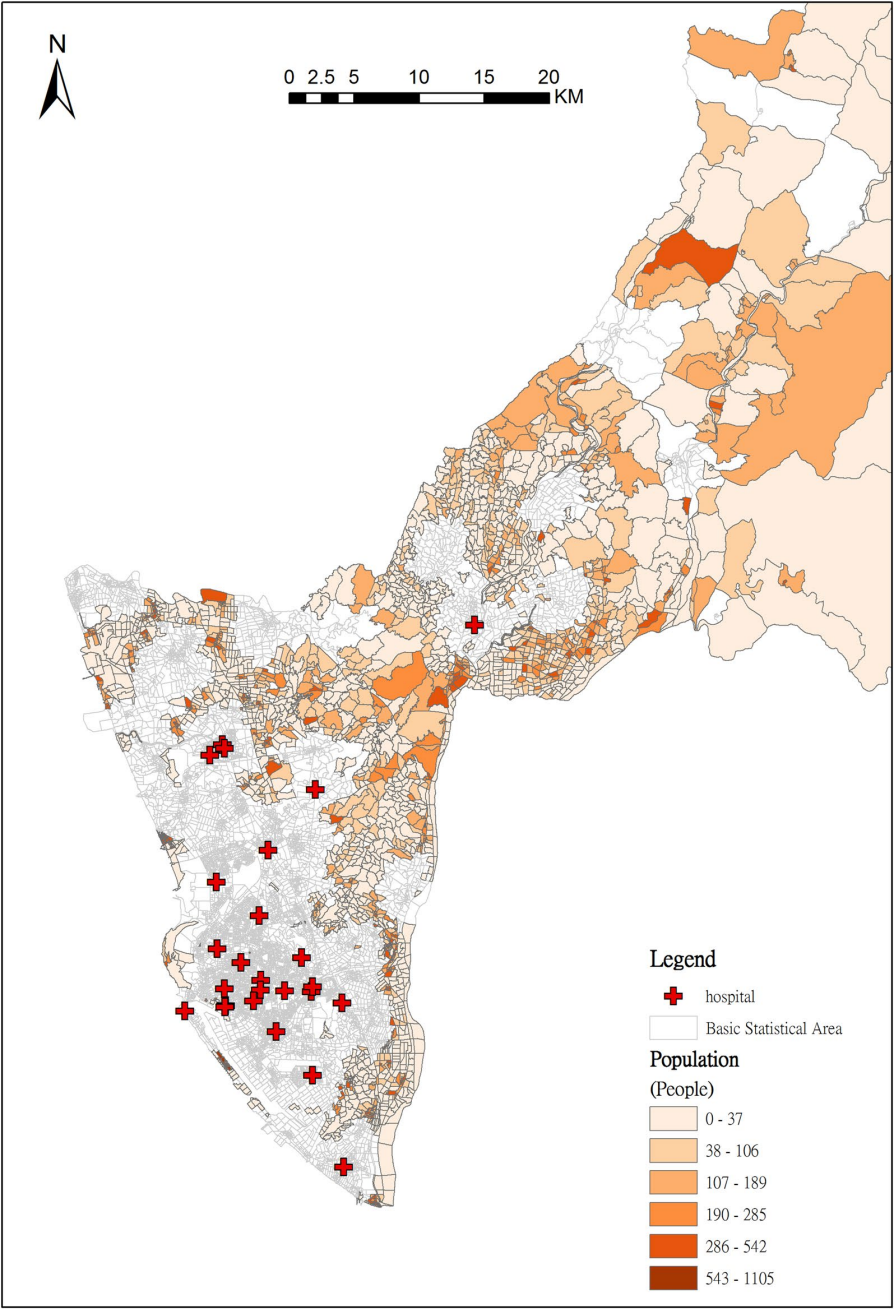
Priority ranking for deployment of AEDs according to the population count in BSA with zero accessibility

After 2SFCA, the priority can be ranked according to the accessibility value. There are 2772 BSAs with zero accessibility. For priority ranking, the location of hospitals is considered. There are 247 BSAs for which transportation time from the BSA centroid to a hospital is within 4 min. Because the population size is the major consideration in deployment of EMS stations, the population is adopted for second priority ranking in the BSAs for which the transportation time from the BSA centroid to a hospital is over 4 min. The higher the population is, the higher the ranking is. The result of ranking is shown in Fig. 7. The highest priority for AED locations is in northern Kaohsiung and marked with dark brown color.

## Discussion

This paper presents a two-stage modeling framework including spatial accessibility evaluation and priority ranking to identify the priority area for locating AEDs. Spatial accessibility is widely used in finding optimal locations for health facilities [23, 43–45]. Both demand and supply are involved in facility location analysis. From the demand side, AEDs should be placed in high OHCA incidence areas [46]. In the traditional approach, we can only analyze the spatial clustering pattern of OHCA events and correlate them with certain risk factors separately [16]. However, there is no single factor which could identify a suitable AED location [15]. Therefore, multiple demographic characteristics are often considered in risk models [28, 47]. In addition to using demographic characteristics, the land use data used in this study, which provides a direct spatial relationship between the classification of land use and the distribution of OHCA events, can help us understand the community structure of OHCA incidents. Our proposed method can then compute one composite indicator from multiple criteria including spatial and non-spatial factors based on their spatial distribution. The indicator is a composite value rather than a single factor, and is utilized as a demand factor in the evaluation of spatial accessibility.

The limited resources may not fulfill the demand in areas with the highest risk, therefore a set of locations is usually selected for solving the location problem [48, 49]. From the supply side, the impact on OHCA survival with the use of onsite or dispatched AED has been discussed in one previous study [50]. In our study, both onsite (existing AEDs) and dispatched (ambulance from EMS stations) AED are considered in the 2SFCA to evaluate spatial accessibility. Thus, all of the supply sites in the communities and public places can be considered simultaneously for evaluating spatial accessibility with our approach in the future.

According to the type of supply location, the service areas of onsite and dispatched AED are different, as suggested by Yang et al. [25]. Within a large service area, the accessibility varies depending on the selection of the decay function, since the catchment size covers many demand locations and other supply locations [24]. In contrast, there is no significant difference in accessibility with a decay function in a small service area. In our study, the mean distance between onsite AED and the center of BSA is about 140 m based on the catchment area of onsite AED, so that the service area only contains the close neighbor BSAs. Thus, the decay function has more influence on accessibility in a dense area than in a sparse area.

AED deployed in public areas such as casinos [51], airports [11] and convenience stores [48] have been shown to be an efficient use of resources for rescuing OHCA patients [28]. In Taiwan, the DOH has stepped up its plan to make AEDs available in public places nationwide. However, our study found that OHCAs frequently occur in housing areas of Kaohsiung city. Likewise, international studies have also observed that the majority of OHCAs occur in residential areas [15, 16, 52]. Thus, the deployment of AEDs in communities is an important issue in the overall emergency medical services system.

Disparity in the coverage of emergency medical service has been found between urban and rural areas around the world [53]. Likewise, a significant difference was also shown in Kaohsiung City. From the perspective of spatial accessibility, we found that the disparity in the deployment of AEDs did not only occur between urban and rural areas. In southwestern Kaohsiung City, the subsets of metropolitan areas with lots of AEDs are scattered, which is marked with blue color in Fig. 6. This means that the supply is much greater than the demand, because many AEDs are concentrated in those areas. To eliminate the disparity and achieve equity, the analysis of spatial accessibility has to be involved in the strategy of AED deployment.

Aside from the disparity in urban areas, the deficiency and unevenness in rural areas of Kaohsiung City are even more serious than those in urban areas. The result shows that the spatial accessibility is low in rural areas because the number of deployed AED is small and the response time is longer. These areas are marked with red color (Fig. 7). The response time is an important factor related to survival rate in OHCA patients, especially in rural areas [54, 55]. To reduce the response time, previous studies have examined advanced life support by aeromedical teams [56], and the use of AED by police officers [57] or by family members [58]. Likewise, the deployment of AED in rural areas is beneficial for OHCA patients in rural areas [59, 60], since the AED placement can extend to a wider EMS coverage and cut rescue response time to improve probability of survival.

Based on an analysis of spatial accessibility, the AED can be deployed in BSAs prioritized from low to high accessibility. The BSA was the smallest statistical unit in the study area, and represented an average of 300 people in each unit. The use of BSA can reduce the spatial unit down to the community level, and help us to identify the AED locations more precisely. Our study shows that a large number of BSAs have low accessibility in Kaohsiung City. This means that the demand for AED, based on historical OHCA incidents, is much greater than the supply of AED. In reality, however, there is not enough money budgeted to provide the required number of AEDs. Under limited finances, the locations of deployed AED have to be efficiently chosen. Utilizing our priority rankings, we can find where the priority BSAs for deploying AEDs are, and the same method of ranking can also be used in each district. Both levels of analysis provide very useful information for government policymakers.

### Limitations

This study had several limitations. First, about 3.1 % of addresses of OHCA events could not be geocoded, due to the incompleteness of the address database. These missing addresses do not influence the result, because the study is concerned with the spatial pattern in clustering of OHCA incidents. To improve the accuracy, commonly used address styles have to be considered in the database, such as intersection and point of interest (POI). Second, registration of installed AED locations is not mandatory, so the number of AED may actually be underestimated. Third, the parameters of response time, based on the speed of both an adult walking and ambulances, will vary under different conditions. In addition, we did not consider the causes of OHCA events such as trauma events, or other demographic characteristics such as gender, income and education [15]. These factors might cause different results for the risk model. The service frequency data of onsite AED cannot be known because it is private property. When the data are released, we can use these data for analysis in a future study.

## Conclusion

The two-stage framework including spatial accessibility evaluation and priority ranking can be beneficial for identifying the local risks and optimizing resource allocation in similar scenarios. The optimized deployment of AEDs can broaden EMS coverage and minimize the problems of the disparity in urban areas and the deficiency in rural areas.

### Availability of data and materials

The cases’ data need IRB approval and apply the data from DOH, Kaohsiung City Government. The land use data need to apply from National Land Surveying and Mapping Center, Ministry of the Interior, Taiwan. The location of AEDs can be directly downloaded from the Taiwan Public AED Registry website of the Ministry of Health and Welfare (http://tw-aed.mohw.gov.tw/). The statistical data contain the total and 65-and-older population of each Basic Statistical Area can be downloaded from the Department of Statistics of Taiwan (http://segis.moi.gov.tw/STAT/).

## Abbreviations

AED: automated external defibrillators
MC2SFCA: multi-criterion two-step floating catchment area
OHCA: out-of-hospital cardiac arrest
EMS: emergency medical service
INLA: integrated nested Laplace approximation
CPR: cardiopulmonary resuscitation
VF: ventricular fibrillation
AHA: American Heart Association
DOH: Department of Health
2SFCA: two-step floating catchment area
E2SFCA: enhanced two-step floating catchment area
V2SFCA: variable two-step floating catchment area
3SFCA: three-step floating catchment area
IRB: Institutional Review Board
EMTs: emergency medical technicians
BSAs: basic statistical areas
POI: point of interest
